# Enhanced detection of pennyroyal essential oil adulteration using FTIR spectroscopy and chemometrics

**DOI:** 10.3389/fchem.2026.1889275

**Published:** 2026-07-16

**Authors:** Abdennacer El Mrabet, Aimen El Orche, Omar Ait El Alia, Morad Kaddouri, Joe Miantezila Basilua, Abdelaaty Abdelaziz Shahat, Rashed N. Herqash, Ibrahim SbaI El Otmani, Mustapha Bouatia, Amal Ait Haj Said

**Affiliations:** 1 Laboratory of Therapeutic Innovation and Artificial Intelligence in Health, Research Team On Pharmaceuticaland Analytical Development, Faculty of Medicineand Pharmacy, Hassan II University of Casablanca, Casablanca, Morocco; 2 Laboratory of the Engineering and Applied Technologies, Higher School of Technology, Sultan Moulay Slimane University, Beni Mellal, Morocco; 3 Department of Biostatistics and Mathematics, Faculty of Pharmacy, Paris Cité University, Paris, France; 4 Department of Pharmacognosy, College of Pharmacy, King Saud University, Riyadh, Saudi Arabia; 5 Center for Research on Medicinal, Aromatic, and Poisonous Plants, DSR, King Saud University, Riyadh, Saudi Arabia; 6 Laboratory of Analytical Chemistry, Team of Formulation and Quality Control of Health Products, Faculty of Medicine and Pharmacy, Mohammed V University in Rabat, Rabat, Morocco

**Keywords:** adulteration, chemometrics, mid infrared spectroscopy, pennyroyal essential oil, quality control

## Abstract

**Introduction:**

The essential oil of pennyroyal has a number of bioactive properties, including antiviral, antifungal, and anti-inflammatory effects. Accurate, rapid, and non-destructive assessment of its quality is therefore a major challenge for the pharmaceutical industry. Conventional methods, such as gas chromatography, although effective, are time-consuming, destructive, and require the use of costly and polluting reagents. In contrast, mid-infrared spectroscopy, a non-invasive analytical method, provides spectral data while preserving sample integrity.

**Methods:**

In this work, Fourier transform infrared spectroscopy (FT-IR) was coupled with machine learning techniques to detect and quantify the adulteration of pennyroyal essential oil with spearmint essential oil at different levels. Principal component analysis (PCA) was used to explore the samples and better understand the structure of the data. Partial least squares regression (PLSR) was then used to predict the levels of adulteration. Variable selection strategies, such as Interval PLS (iPLS) in forward and reverse modes, were applied to improve model performance and interpretability.

**Results:**

By identifying the most informative spectral regions, these methods improved the accuracy and resilience of the predictive models. The detrend pre-processing method produced the most efficient model, with an RPD of 8.29, an RMSEt of 1.25, and an R^2^t coefficient of determination of 99.47%. Most of the variable selection methods performed remarkably well, whether with forward or reverse iPLS, and regardless of the type of pretreatment used. Cross-validation R^2^ values ranged between 99.0% and 99.95%, underlining their exceptional predictive capacity, with low RMSE values between 0.75 and 1.1.

**Discussion:**

These findings demonstrate how infrared spectroscopy and machine learning may be successfully combined to quickly, sustainably, and accurately assess the quality of pennyroyal essential oil, offering a powerful alternative to traditional chromatographic techniques for industry-level quality control.

## 1 Introduction

According to the International Organization for Standardization (ISO) and the European Pharmacopoeia, an essential oil is a fragrant product with a complex composition, obtained from a botanically defined plant material either by steam distillation, dry distillation, or a mechanical process without heating (El Mrabet et al., 2024). Essential oils often contain more than a hundred chemical compounds, which can be classified into different groups based on their chemical properties and their effects on the human body. There are two main categories: the calming and relaxing category, which includes aldehydes, ketones, and esters, and the stimulating and tonic category, which includes monoterpenes, terpene oxides, phenols, and monoterpenols (Hüsnü et al., 2007). Essential oils are widely used in food, cosmetics, detergents, pharmaceuticals, and pesticides, but their high demand and low yields often lead to economic-motivated adulteration (Tongnuanchan and Benjakul, 2014).

Pennyroyal (*Mentha pulegium*) is a medicinal plant native to Europe, North Africa, and the Middle East (Mahboubi and Haghi, 2008). Its leaves and flowers have long been used in traditional medicine for their antiseptic, antioxidant, and cytotoxic properties (Mahboubi and Haghi, 2008). Its essential oil is mainly composed of pulegone, a compound known for its antioxidant (El-Ghorab, 2006), antimicrobial and anxiolytic effects (Cagliero et al., 2022; Cozzolino, 2009).

However, increasingly frequent reports reveal the adulteration of this essential oil with cheaper substitutes, such as nanaah mint oil, posing an ongoing challenge for the global aromatic and medicinal plant industry (Lafhal et al., 2016).

The approach recommended by the International Organization for Standardization (ISO) for the analysis and quality control of pennyroyal essential oil is based on gas chromatography, either coupled with mass spectrometry (GC-MS) or using a flame ionization detector (GC-FID) (El Mrabet et al., 2024). However, GC-FID is generally preferred for essential oil analysis due to its high sensitivity to most organic compounds present in essential oils. It enables the detection of even minor volatile constituents, which is essential for establishing a reliable chemical profile and assessing the quality of the essential oil (Cagliero et al., 2022).

Pennyroyal essential oil must comply with the requirements of AFNOR NFT 75-216, which sets specific criteria for the content of volatile compounds to ensure its authenticity and high quality. These criteria are listed in Table 1.

**TABLE 1:** Pennyroyal essential oil ISO standard (AFNOR NFT75-216).

Compound	Average content (%)
Pulegone	75%–90%
Menthone	0.2%–3%
Isomenthone	2%–3%
Neomenthol	< 5%
Caryophyllène	< 2%
Limonene	< 1.5%

However, this technique is destructive, meaning that the samples cannot be reused after analysis. In addition, it requires the use of costly and environmentally harmful reagents, as well as the expertise of trained analytical chemists to carry out the analysis properly (El-Ghorab, 2006; Flamini et al., 1999; Hüsnü et al., 2007).

In order to provide reliable, rapid, and non-destructive methods that do not require sample preparation, spectroscopic techniques such as infrared spectroscopy, UV-Visible spectroscopy, and Raman spectroscopy, combined with chemometric tools, have already been employed (Ait et al., 2026a). These methods not only enable the detection of adulteration but also allow the prediction of its percentage, as well as the proportion of volatile compounds present in essential oils (Ait et al., 2026b; Jayasekher et al., 2024; Johnson et al., 2022; Kanakis et al., 2012).

Only a few studies have used infrared spectroscopy and chemometrics to assess the quality and authenticity of pennyroyal essential oil, while the majority of research focuses on gas chromatography and the measurement of physico-chemical parameters (Kucharska-Ambrożej et al., 2021).

This study therefore aimed to use MIR spectroscopy as a rapid analytical tool to develop chemometric models (PCA, PLSR) to effectively discriminate between pure and adulterated samples and to accurately quantify the percentage of adulteration of pennyroyal essential oil by spearmint.

Additionally, three variable selection algorithms Backward IPLS, Forward IPLS, and siPLS were applied to identify the most informative spectral regions. This approach allowed for the optimization of the developed models by eliminating redundant or uninformative variables while maintaining high predictive performance.

### 1.1 Literature review

The application of mid-infrared (MIR) spectroscopy in quality control has gained significant attention in recent years, mainly due to advances in Fourier-transform instruments and improved sample presentation systems (Cuny, 2008). From 2004 to 2024, approximately sixty studies have investigated the use of infrared spectroscopy combined with chemometric tools for essential oil quality assessment. These works primarily focus on detecting adulteration, often involving the addition of cheaper essential oils or vegetable oils with similar chemical compositions, as well as predicting adulteration levels using partial least squares regression (PLSR). Other studies have addressed the classification of essential oils based on geographical origin, botanical variety, or the prediction of volatile compound concentrations using chemometric models. In most cases, gas chromatography data are correlated with infrared spectral fingerprints (near- or mid-infrared) to develop predictive statistical models.

Vibrational spectroscopy techniques have also been widely explored for essential oil analysis. Early studies demonstrated the potential of Raman and FT-Raman spectroscopy, combined with density functional theory (DFT), to identify major components of eucalyptus essential oils and study their distribution within plant tissues (Baranska et al., 2006). These approaches highlighted the ability of vibrational spectroscopy to provide rapid and reliable compositional information consistent with gas chromatographic results.

Subsequent research has confirmed the effectiveness of MIR spectroscopy combined with chemometrics for essential oil authentication and classification. For example, chemometric analysis of MIR data successfully differentiated Lavandin essential oils from different geographical origins and quantified major chemical constituents, producing results comparable to gas chromatography while offering faster analysis (Bombarda et al., 2008). Similarly, MIR and near-infrared (NIR) spectroscopy have been shown to effectively classify plant species and predict essential oil composition, with partial least squares (PLS) models providing high predictive accuracy (Sandasi et al., 2010; Tankeu et al., 2014).

Further studies have demonstrated the applicability of vibrational spectroscopy for quality control in various essential oils, including lavender, lavandin, buchu, and encapsulated oils. These approaches have shown strong correlations between spectroscopic data and chromatographic reference methods, confirming their suitability for routine analysis (Rodríguez et al., 2018). In addition, FT-MIR spectroscopy has been successfully applied for botanical authentication and detection of mislabelled or adulterated essential oils, particularly when combined with classification models such as SIMCA (Ordoudi et al., 2020).

More recently, integrated spectroscopic and chemometric approaches have been applied to complex authentication problems. Studies combining FT-MIR, Raman spectroscopy, and gas chromatography have demonstrated high accuracy in distinguishing authentic from adulterated samples, as well as in classifying essential oils according to species and chemotype using multivariate techniques such as principal component analysis (PCA), hierarchical cluster analysis (HCA), and partial least squares discriminant analysis (PLS-DA) (Cebi, Arici, and Sagdic, 2021; Truzzi et al., 2022).

Overall, these studies confirm that vibrational spectroscopy, particularly MIR combined with chemometric tools, represents a fast, non-destructive, and cost-effective alternative to conventional chromatographic techniques for essential oil analysis. However, despite the large number of studies available, relatively few have focused on specific essential oils such as pennyroyal, highlighting a clear gap in the literature and the need for further research in this area.

## 2 Materials and methods

### 2.1 Preparation of authentic and adulterated pennyroyal oil samples

Pennyroyal flowers were collected from the Tetouan and Tangier regions, while spearmint leaves were obtained from Fez. The geographic coordinates of the sampling sites are reported in Table 2. Following hydrodistillation using a Clevenger apparatus for 3 h, adulteration was performed by blending pennyroyal essential oil with spearmint essential oil at 20 different concentrations (1%–50%). For each concentration level, three independent mixtures were prepared, yielding 60 adulterated samples. Additionally, 40 pure essential oil samples (20 pure pennyroyal and 20 pure spearmint) were included as controls, resulting in a total dataset of 100 samples.

**TABLE 2:** Sample details.

The province	The plant	Region	Height (m)	Latitude (N)	Longitude (W)
Tetouan	Pennyroyal	Tangier – Tetouan	57	35° 34′ 21″	5° 21′ 17″
FES	Spearmint	Fes- meknes	407	34° 01' 59.27″	−5° 00' 1.01″

### 2.2 FT-MIR spectroscopy

Infrared spectroscopy is a vibrational spectroscopic technique used to characterize the molecular composition of substances. It is based on the absorption of a light beam by the sample, with radiation distributed across three regions: near-infrared, mid-infrared, and far-infrared. This absorption mainly induces stretching and bending vibrations (Tarhan et al., 2023).

Spectra were recorded in ATR mode (Shimadzu FTIR, Xross IR, S/N A229360) by applying 20 μL of sample directly onto the crystal, with a spectral resolution of 4 cm^-1^ and 30 scans per measurement; the ATR cell was cleaned with isopropanol and dried after each acquisition.

The ATR mode is preferred for the analysis of essential oils because it does not require any prior sample preparation. A single drop deposited on the crystal is sufficient for analysis, which is a significant advantage for expensive and rare samples (El Maouardi et al., 2023; Liu et al., 2024; Mahboubi and Haghi, 2008).

In order to eliminate undesirable effects that may alter the quality of the obtained spectra, such as photon scattering and measurement noise, spectral preprocessing techniques were applied to the results obtained from infrared spectroscopy.

Savitzky–Golay differentiation was applied to correct baseline and band overlap effects (Truzzi et al., 2021). MSC and SNV reduced scattering and multiplicative interferences, while the detrend method minimized photon scattering and signal loss (Yamashita et al., 2021).

### 2.3 Gas chromatography analysis

Our samples were analyzed using gas chromatography coupled with a flame ionization detector (GC-FID), a technique that enables the identification and quantification of volatile compounds. The obtained results were compared to the standards established by ISO.

The analysis of the extracted essential oil was carried out using a gas chromatograph (Agilent HP6850 Technology system) equipped with a flame ionization detector and a DB-WAX column (20 m × 100 μm). The oven temperature program included a linear ramp from 60 °C to 248 °C at a rate of 12 °C/min. Hydrogen was used as the carrier gas at a flow rate of 0.7 mL/min.

### 2.4 Multivariate data analysis methods

After collecting the experimental data, the analysis relies on unsupervised methods such as PCA to explore the samples (Petrakis et al., 2009; Rached et al., 2025) and on supervised methods such as PLSR to model and predict the relationships between the spectral data and the known percentages of adulteration (Reda et al., 2020; Rutledge et al., 2021; Tarhan et al., 2023; Taylan et al., 2021; Tongnuanchan and Benjakul, 2014).

To improve the accuracy of the results obtained by PLS regression, variable selection methods have been applied, in particular progressive IPLS, backward IPLS. These techniques involve dividing the full spectrum into multiple wavelength intervals and then building a separate PLS model for each interval (Xie et al., 2006; Yamashita et al., 2021). The goal is to identify the spectral regions that contain the most relevant information for predicting the dependent variable of interest. Thus, these approaches help to eliminate non-informative variables while preserving the predictive ability of the model.

iPLS strategies tested involve either progressively building the model by adding the most relevant variables (forward iPLS) or simplifying the model by gradually removing the least significant variables (backward iPLS) (El Mrabet et al., 2024; Reda et al., 2020).

Predictive accuracy was evaluated through internal and external validation. The entire dataset (100 samples) was divided into a calibration set (75%, n = 75 samples) and an independent external validation set (25%, n = 25 samples). For internal validation, a K-fold cross-validation strategy was applied to the calibration set by dividing the data into K subsets, iteratively using K-1 folds for training and one fold for validation. To more rigorously test the model’s robustness against entirely independent matrices, the external validation set (25%) included ten new samples of pure Mentha pulegium purchased from a pharmacy in Fez adulterated with spearmint essential oil at various concentrations—alongside independent batches from the initial series. The model’s performance was evaluated using R^2^ (Coefficient of Determination), RMSE (Root Mean Square Error), and RPD (Residual Prediction Deviation). The predicted values were compared to the reference values to assess the model’s actual predictive capability and ensure the absence of overfitting.

### 2.5 Software

Data analysis and visualization were carried out using the R programming language (v 4.5.0).

## 3 Results and discussion

### 3.1 Chromatographic results

The Table 3 below presents the analysis results of pennyroyal essential oil obtained by gas chromatography (GC). The chromatographic profile reveals a strong predominance of pulegone, present at over 80%, which clearly confirms that the essential oil is from Mentha pulegium. Neomenthol, a monoterpene alcohol with a refreshing effect, is the second most abundant compound at 1.82%. It is followed by α-humulene, a sesquiterpene with a woody aroma, present at 1.07%. According to the AFNOR NF T75-216 standard, this pennyroyal essential oil complies with the established quality requirements, our results are consistent with those of Sara Rached et al., who reported that the essential oil of pennyroyal mint from the Zaer region (near Rabat, western Morocco) is dominated by pulegone, which accounts for 80.03% of its composition (Rached et al., 2025). On the basis of the results obtained from the gas chromatography analysis of spearmint essential oil, which are grouped together in Table 4, carvone is identified as the major compound in spearmint essential oil, accounting for 52.43% of the composition. This compound, which accounts for more than half of the oil’s content, is considered to be the main chemical marker of spearmint. It is followed by limonene (8.98%), which contributes a citrus note, as well as myrcene (3.43%) and 1,8-cineole (3.75%), the latter contributing to the characteristic sensation of freshness. Other compounds, although present in smaller quantities, such as α-pinene, β-pinene and carveol, also play an important role in the overall aroma and biological properties of spearmint essential oil.

**TABLE 3:** Analysis of the chemical constituents of pennyroyal EO samples using GC-FID.

Compound	Contents %
Pinene (alpha)	0.40
Pinene (beta)	0.30
Sabinene	0.05
Limonene	0.88
para mentha-3,8-diene	0.24
Octanol 3	0.81
Neomenthol	1.82
Menthone	0.30
Caryophyllene beta	0.62
Humulene (alpha)	1.07
Pulegone	81.49
Germacrene D	0.03
Piperitone	0.05

**TABLE 4:** GC-FID analysis of chemical constituents in spearmint EO samples.

Compound	Contents %
*pinene (alpha)*	1.58
*Myrcene*	3.43
*Limonène*	8.98
*Carvone*	52.43
*carveol(trans)*	0.27
*1,8 cineole*	3.75
*pinene (beta)*	1.13
*caryophyllene béta*	1.26

The results obtained by gas chromatography are consistent with the study conducted by Nadia Zekri et al., who reported similar findings, with minor percentage variations that can be attributed to differences in geographical origin. In their study, carvone was identified as the major compound, followed by limonene, with proportions of 54.79% and 9.69%, respectively (Middle et al., 2023).

### 3.2 FT- MIR spectroscopy measurement

**FIGURE 1:**
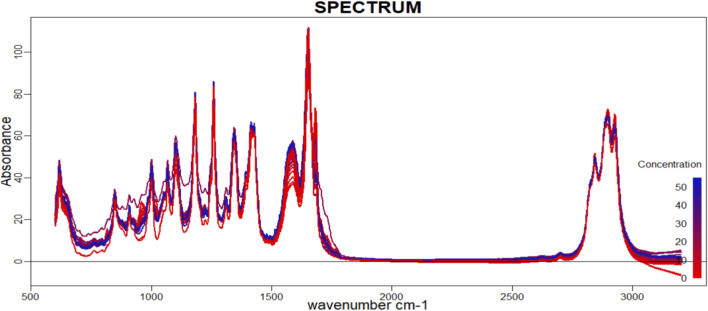
Spectroscopic analyses FT-MIR of the essential oil of pennyroyal falsified with spearmint.

The following Figure 1 Illustrates the characteristic infrared spectra of pennyroyal essential oil adulterated with spearmint. These spectra reveal the main absorption bands corresponding to the major compounds of pennyroyal oil, dominated by the presence of pulegone. For example, the intense bands observed at 2970 cm^-1^ and 2860 cm^-1^ are attributed to the symmetric and asymmetric stretching vibrations of the C–H, CH_2_, and CH_3_ bonds, characteristic of pulegone and α-humulene (Petrakis et al., 2009).

A very intense band also appears around 1700 cm^-1^, indicating the stretching of the C=O bond of the carbonyl functions present in monoterpenic ketones such as pulegone and carvone. On the other hand, a moderate intensity band around 1600 cm^-1^ is associated with the presence of unsaturated cycles, particularly those of carvone and limonene, typical signatures of spearmint oil used as an adulterant (El Maouardi et al., 2023; Petrakis et al., 2009).

The bands located at 1450 cm^-1^ and 1470 cm^-1^ correspond to the bending vibrations of the methyl (CH_3_) and methylene (CH_2_) groups, while the one observed at 1370 cm^-1^ can be attributed to a symmetrical bending of the methyl group. Finally, the region between 1000 cm^-1^ and 1300 cm^-1^ shows several bands associated with the bending vibrations of the C–O bond in ether functions, particularly those of pulegone and carvone, with maxima observed around 1170 cm^-1^ and 1310 cm^-1^ (Kanakis et al., 2012).

Similar FT-IR based analyses have been previously applied to pennyroyal essential oil, demonstrating spectral regions associated with major volatile constituents such as pulegone and supporting the use of FT-IR for chemical characterization of Mentha pulegium oils (Petrakis et al., 2009).

### 3.3 Classification of pure and adulterated essential oil of pennyroyal

**FIGURE 2:**
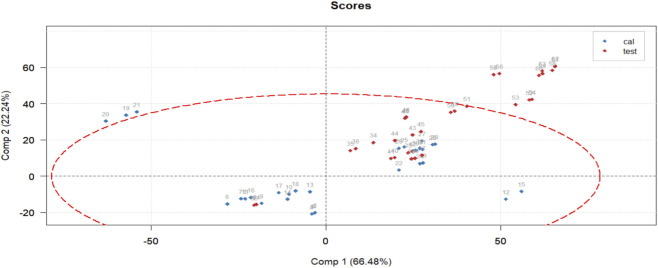
Scores plot of PCA model with two PCs for FT-MIR data.

Application of principal component analysis to the results obtained by infrared spectroscopy (Figure 2) showed that The first principal component explains the major part of the total variance of the data (66.48%) while the second principal component explains another significant proportion (22.24%). Generally, these two components explain 88.27% of the total variance, which is excellent for the data. PCA revealed that there are at least two distinct groups: the first is concentrated around the center and the second is more dispersed along axis 1 towards the ends (Samples 19, 20, 21 on the left and 55, 56, 62 on the right). The samples outside the ellipse can be considered atypical values (in fact, these are the samples highly falsified by 40%–50%).

The application of PCA to the results obtained by FT-IR demonstrated considerable efficiency in detecting atypical values in spectroscopic data and identifying potential frauds that may pose a risk to human health. These findings are consistent with those of Liu et al. (Liu et al., 2024), who highlighted the importance of PCA for improving data quality and detecting atypical values in spectroscopic datasets (Liu et al., 2024).

### 3.4 Quantification of the percentage of adulteration in the essential oil of pennyroyal

In order to develop a simple and efficient analytical approach for estimating the adulteration rate of pennyroyal essential oil, the correlation between the adulteration percentages of laboratory prepared samples and their infrared spectra was modeled using partial least squares regression (PLS). To improve the quality of the spectra and reduce undesirable effects that could distort them, such as photon scattering or electronic noise, statistical preprocessing techniques were applied, including detrending, standard normal variate (SNV) normalization, and multiplicative scatter correction (MSC).

Seventy-five percent of the data were used for calibration to establish the mathematical relationship between the independent variables (the spectra obtained by FT-IR) and the dependent variable (the reference value of the studied attribute to be predicted), while the remaining 25% constituted the test set, used to evaluate the accuracy and performance of the models, thereby assessing their quality. This evaluation was primarily based on the calculation of the root mean square error for calibration (RMSEc) and for testing (RMSEt), as well as the coefficient of determination for calibration (R^2^c) and for testing (R^2^t). The most effective model is the one that exhibits a high coefficient of determination while maintaining a reasonable number of latent variables.

**FIGURE 3:**
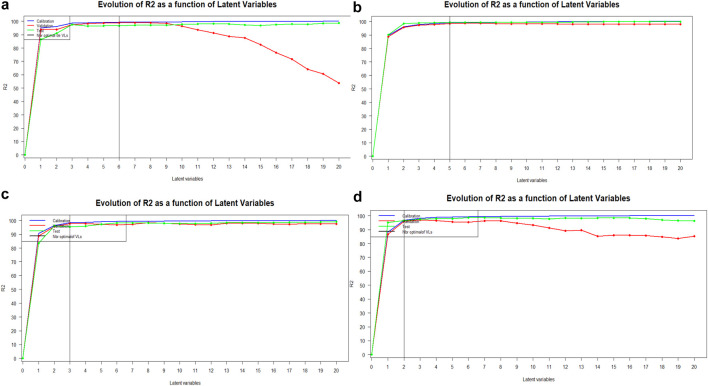
R^2^ variation with the number of latent variables for PLS models using different preprocessing: **(a)** none, **(b)** detrend, **(c)** SNV, **(d)** MSC.

The use of a limited number of latent variables helps to improve the robustness of the model, i.e., its ability to remain stable in the face of external perturbations, but too few can lead to underfitting (Rutledge et al., 2021). To identify the optimal number of latent variables, we therefore examined the evolution of the coefficient of determination (R^2^) as a function of this parameter for each model (Figure 3), whether based on raw data or pre-processed by various methods.

The best performing model was obtained using the data without pre-treatment, with a cross-validation (R^2^cv) of 99.03%, an RMSEcv of 1.46 and six latent variables indicating a good fit of the model developed to the observed data (Table 5), other pre-treatment techniques also produced satisfactory results, with R^2^cv values of between 96.21% and 98.52%, and RMSEcv values ranging from 1.70 to 2.7, demonstrating its potential effectiveness in predicting the rate of adulteration of essential oil of pennyroyal.

**TABLE 5:** Performance metrics for the prediction of pennyroyal essential oil adulteration.

Spectral region	Preprocessing	LVs	R^2^ c	R^2^cv	RMSEc	RMSEv
600–4000 cm^-1^	Raw data	6	99.35	99.03	1.19	1.46
	Detrend	5	99.08	98.52	1.35	1.72
	SNV	3	98.67	98.15	1.72	2.05
	MSC	2	96.82	96.21	3.02	2.77

These results highlight the importance of FT-IR spectroscopy for accurately predicting the adulteration percentage of essential oils. They are consistent with the findings of (Truzzi et al., 2021), who showed that ATR-FTIR coupled with PLS regression could quantify the adulteration of essential oils such as lavender and citronella with very high coefficients of determination (R^2^c = 0.997 and R^2^cv = 0.996) and low RMSE values, thus confirming the reliability of this analytical approach for detecting adulteration (Truzzi et al., 2021).

### 3.5 External validation

**FIGURE 4:**
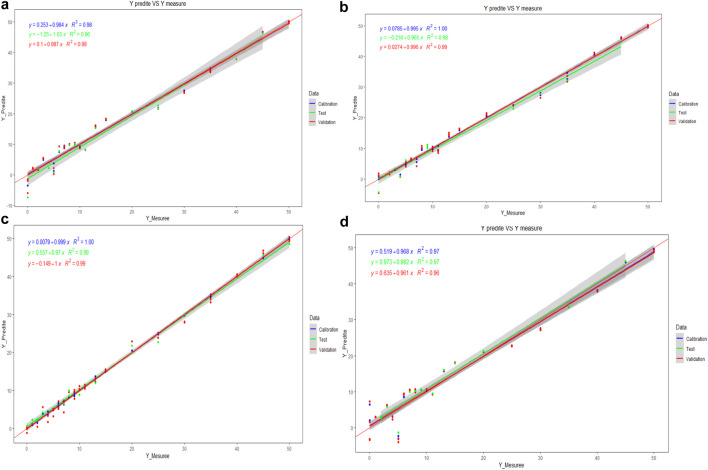
PLSR models developed using different preprocessing methods: **(a)** raw data, **(b)** detrend method, **(c)** SNV preprocessing, and **(d)** MSC preprocessing.

To evaluate the predictive performance of the developed models and their ability to generalize to new samples, external validation was carried out using an independent set of ten samples from a new geographical region (Fes). Predictions were made solely based on their infrared spectra and then compared with the reference values. The results of this validation are presented in Figure 4, showing a strong correlation between the predicted values and the reference values, with a linear distribution around the regression line. This indicates good agreement between the predictions obtained by the models developed and the experimental measurements, whatever the pre-treatment technique applied. The best-performing model was obtained by applying the detrend preprocessing method, with a test coefficient of determination (R^2^t) of 99.47%, a test root mean square error (RMSEt) of 1.25, and a correlation coefficient of 99.54%. These indicators demonstrate the model’s robustness and reliability (Table 6). Other preprocessing methods, including the Savitzky-Golay algorithm, were also evaluated but showed lower predictive performance than the detrend technique.

**TABLE 6:** Performance standards for outside verification.

Spectral region	Preprocessing	R^2^t	RPD	AIC	BIC	Correlation	RMSEt
600–4000 cm^-1^	Raw data	98.57	11.16	2772	5326	99.94	1.85
	Detrend	99.47	8.29	2868	5422	99.54	1.25
	SNV	98.73	13.08	2763	5286	99.67	1.86
	MSC	96.56	5.19	2939	5493	98.39	2.44

These results are consistent with those reported in the literature. For example, in a recent study, PLSR models based on ATR-FTIR achieved coefficients of determination greater than 0.99 in both calibration and validation, with very low RMSEP, demonstrating that the predicted values were very close to the reference values for quantifying adulteration in essential oils. This confirms the reliability of the FT-IR approach coupled with PLS regression for detecting and quantifying adulteration in such matrices (Jayasekher et al., 2024).

In addition, the performance/deviation ratio (PDR) was used as an additional evaluation criterion. A RPD value greater than 3 is generally considered to be the threshold at which a model is considered reliable for prediction. In this study, all the models developed had a PDS greater than 3, confirming their accuracy and relevance for predictive applications.

## 4 Prediction of adulteration of spearmint oil using the interval partial least squares (IPLS forward, IPLS backward) methods

**FIGURE 5:**
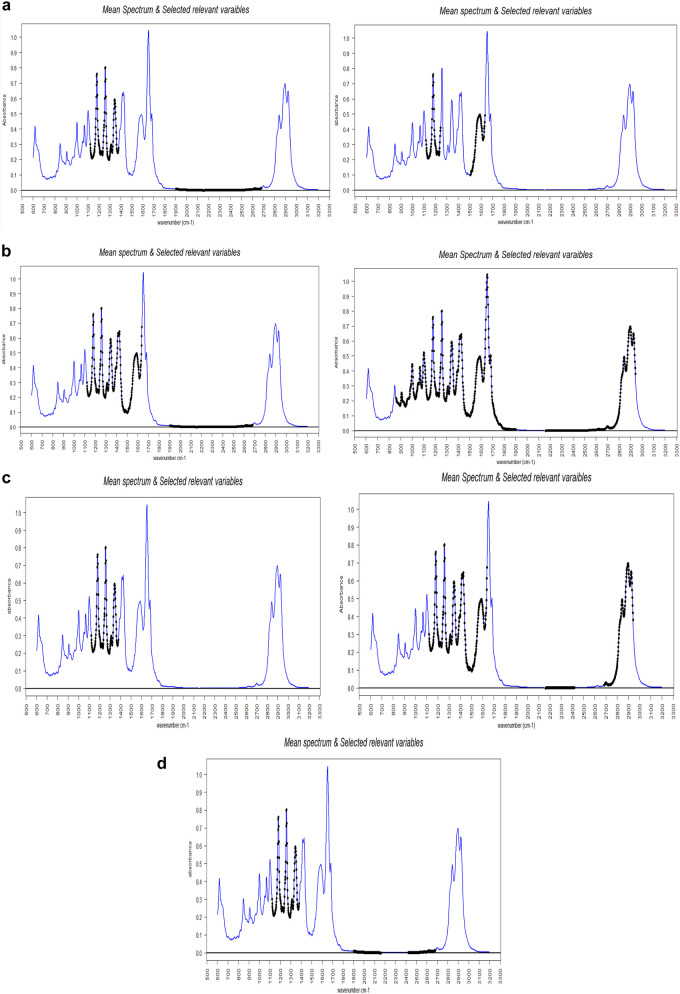
Wavenumber selected by IPLS (backward, forward) respectively to predict the percentage of adulteration. **(a)** Raw data, **(b)** Detrend, **(c)** SNV, **(d)** MSC.

In order to optimise the models developed by partial least squares regression (PLSR) while eliminating uninformative variables from the spectrum and retaining its predictive capacity, several variable selection methods were applied, including iPLS backward, iPLS forward. The results obtained using these techniques are presented in Table 7 and Figure 5.

**TABLE 7:** Overview of the variable selection methodology using several types of IPLS.

Method	LVs	R^2^cal	R^2^val	RMSEcal	RMSEval	Nvar	RPD
IPLS (forward)	11	99.5	99.5	0.57	0.75	134	20
IPLS (backward)	10	99.5	99.5	0.69	0.99	540	29
IPLS forward detrend	10	99.5	99.5	0.7	0.96	675	15.8
IPLS backward detrend	10	99.5	99.5	0.72	1.05	945	15
IPLS forward SNV	9	99.5	99	0.83	1.07	135	14.2
IPLS backward SNV	10	99.5	99	0.76	1.1	540	14
IPLS forward MSC	10	99.5	99.5	0.77	1.03	405	14.7

Most of the variable selection techniques offer excellent performance, whether for progestational IPLS or for retrograde IPLS using any of the preprocessing techniques with R^2^val values that vary between 99.5 and 99 underlining its exceptional predictive capacity, and a low RMSEval value that varies between 0.75 and 1.1 and a number of latent variables that varies between 9 and 11but the significant difference between these models is the number of variables selected in each model, the one that selects the lowest number of variables is IPLS forward without data preprocessing with a number of variables that equals 134 while the model developed by IPLS backward using the detrend method selected the highest number of variables 945 variables.

Furthermore, the Performance to Deviation Ratio (PRD) must be greater than 3 for a model to be considered as having good predictive accuracy. All the models developed in this study have a PDR above this threshold, which indicates that they all have satisfactory or even excellent predictive quality.

The majority of the variables selected by the iPLS algorithms are concentrated in the spectral region from 1100 to 1700 cm-^1^. This region appears to play a key role in the detection of adulteration of spearmint essential oil. In particular, intense bands around 1680 cm-^1^ are attributed to elongation of the C=O bond of the carbonyl function, characteristic of pulegone and carvone. These two compounds represent the majority constituents, respectively, of pure spearmint oil and adulterant oil, which explains the relevance of this spectral region for differentiating the samples. In addition, bands located at 1450 and 1370 cm-^1^ correspond to the deformation vibrations of the methylene (CH_2_) and methyl (CH_3_) groups, present in particular in pulegone, limonene and carvone. These signals reinforce the discriminating capacity of this zone. This specific portion of the FT-MIR spectrum therefore appears to be an essential chemical marker, enabling pure samples to be effectively distinguished from adulterated samples.

## 5 General discussion

All the volatile compounds identified in pure pennyroyal essential oil during this study are consistent with the data reported in the literature (Petrakis et al., 2009). Given the increasing number of cases of falsification on the essential oil market, in particular the dilution of spearmint oil with cheaper alternatives such as spearmint oil, the results obtained using MIR spectroscopy combined with chemometric techniques offer a promising solution for ensuring the authenticity and quality control of essential oils.

Our results are consistent with previous work, including the study by Osman Taylan et al. (2021), which reported a 99.99% predictive R^2^ for the quantification of adulterations in commercial samples of peppermint oil adulterated with L-menthol and spearmint.

However, unlike these studies, our research focused specifically on quantifying the levels of adulteration in pennoroyal oil, using spearmint oil as the adulterant, due to its physicochemical and aromatic characteristics similar to those of spearmint.

Despite these advantages, certain limitations should be noted. The specificity to experimental conditions is an important factor: a model calibrated on spectra obtained in a given laboratory (instrument type, temperature, humidity, spectral resolution) may lose accuracy when applied in a different experimental context. Additionally, the size and representativeness of the samples play a crucial role: limited sampling can reduce the model’s ability to generalize accurate predictions, especially when testing the model on samples from new batches.

## 6 Conclusion

In Morocco, the essential oil production sector is increasingly regarded as a strategic lever for socio-economic development across various regions. In this context, the modernization of the sector and the improvement of quality control methods particularly to combat fraud have been identified by the Moroccan authorities as key priorities to ensure sustainable growth and international competitiveness.

This study highlights the potential of infrared spectroscopy for detecting the adulteration of pennyroyal essential oil with spearmint oil. The combined use of infrared spectroscopy and chemometric techniques proves to be a powerful approach, enabling the detection of even low levels of adulteration and offering a fast, reliable, and cost-effective method for essential oil quality control.

The developed models successfully detect and predict the percentage of adulteration in pennyroyal essential oil. However, applying this approach to other types of essential oils will require the development of specific models tailored to the unique chemical characteristics and potential adulterants of each oil.

## Data Availability

The raw data supporting the conclusions of this article will be made available by the authors, without undue reservation.
